# Supplementary material for: Vibration hotspots reveal longitudinal funneling of sound-evoked motion in the mammalian cochlea
Source: Nat Commun. 2018 Aug 3;9:3054. doi: 10.1038/s41467-018-05483-z (PMC6076242; doi:10.1038/s41467-018-05483-z)
Supplement: Supplementary file 1 — Supplementary Information [file 41467_2018_5483_MOESM1_ESM.pdf]

Supplementary Information

Vibration hotspots reveal longitudinal funneling of sound-evoked motion in the mammalian cochlea

Cooper et al.

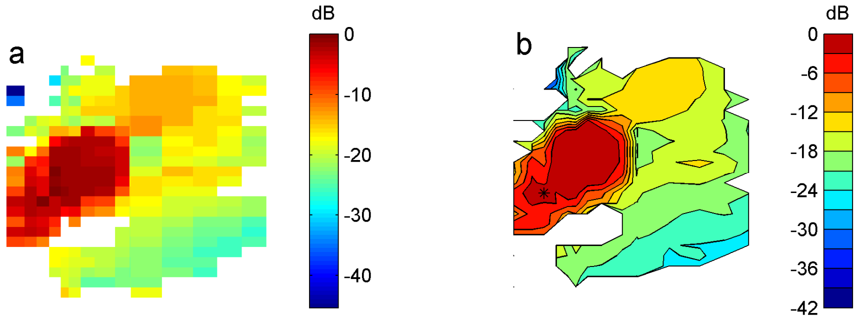

Supplementary Figure 1. Vibration mapping technique. **(a)** Vibration magnitudes plotted as color-coded "pixels" form a two-dimensional spatial map across the width and depth of the cochlear partition. This particular map is based on measurements made at 476 loci, obtained by combining data from 17 positions of the OCT beam, each with a 'vertical' resolution of ~8 µm. Loci providing non-significant vibration estimates are shown as white spaces in the figures. **(b)** Two-dimensional interpolation permits the derivation of iso-amplitude contours within the field of measurement**.** No smoothing of these contours is performed. Color codes indicate vibration magnitudes (mapped on a logarithmic scale) relative to the maximum observed vibration (in this case 3 nm). The location of the maximum is marked by the asterisk in B. Preparation RG16725, CF 22.6 kHz (see Fig. 2 of main text).

Supplementary Note 1: Interpolation method for the vibration maps

Supplementary Fig. 1 illustrates the effects of two-dimensional interpolation in the derivation of a spatial vibration map. The measurement technique provides vibration estimates at all depths along each measurement pathway (Supplementary Fig. 1a). Only those estimates that exceed a statistically defined noise-floor are used for subsequent analysis (see Methods). The results of interpolating these data and fitting contours at 3 dB intervals (as used throughout the current paper) are shown in Supplementary Fig. 1b. Note that the construction of contour maps from the pixelated data involves no form of smoothing or averaging.

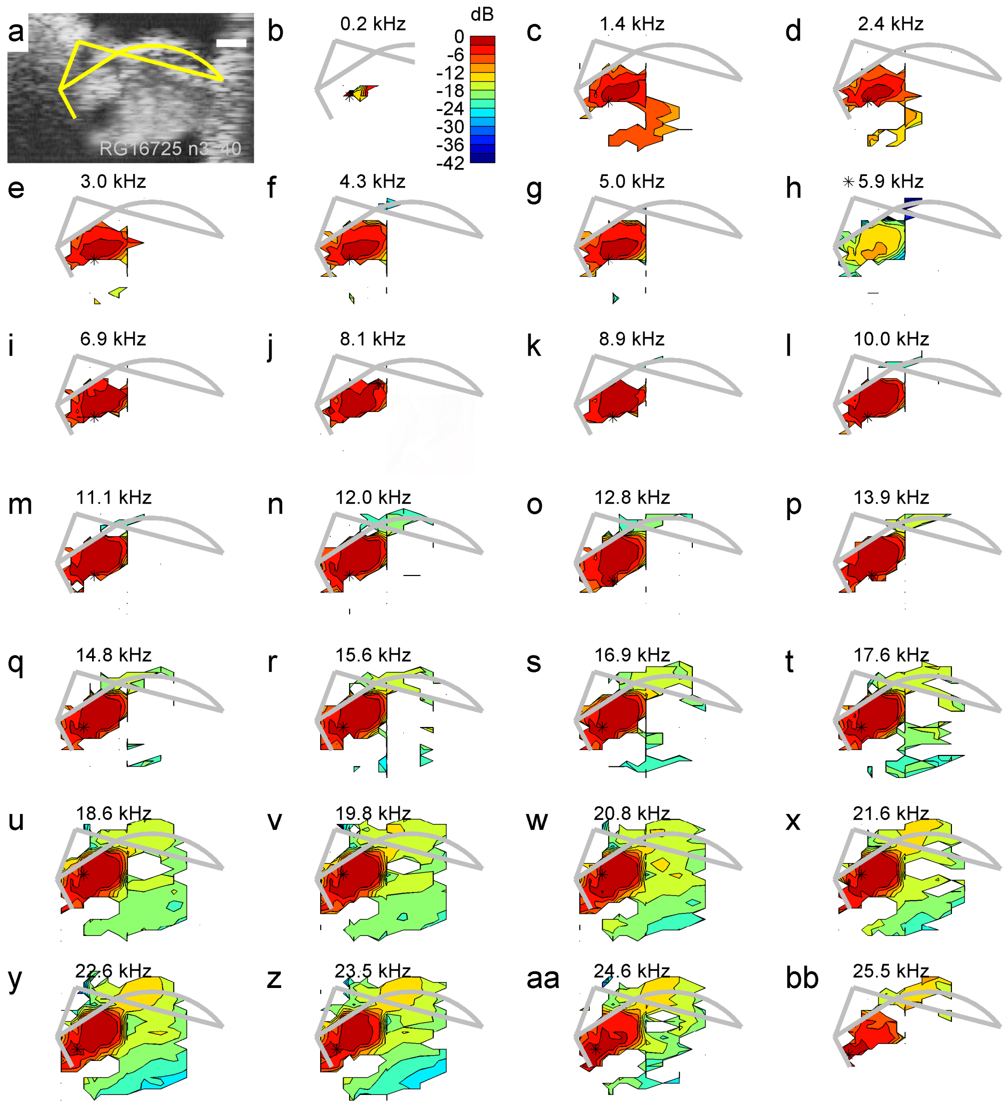

Supplementary Figure 2. Maps of vibration magnitude for acoustic frequencies spanning 0.2-25.5 kHz (panels **a-bb**) in the same preparation illustrated in Figs. 2 and 3. All stimulus components were presented simultaneously at 40 dB SPL, and their responses were isolated by Fourier analysis. Scale bar in panel a, 25 μm. Color code (legend in panel b) indicates vibration magnitude normalized to the peak magnitude for each frequency. The location of the peak is marked by an asterisk. CF was 22.6 kHz (see Fig. 2 of main text).

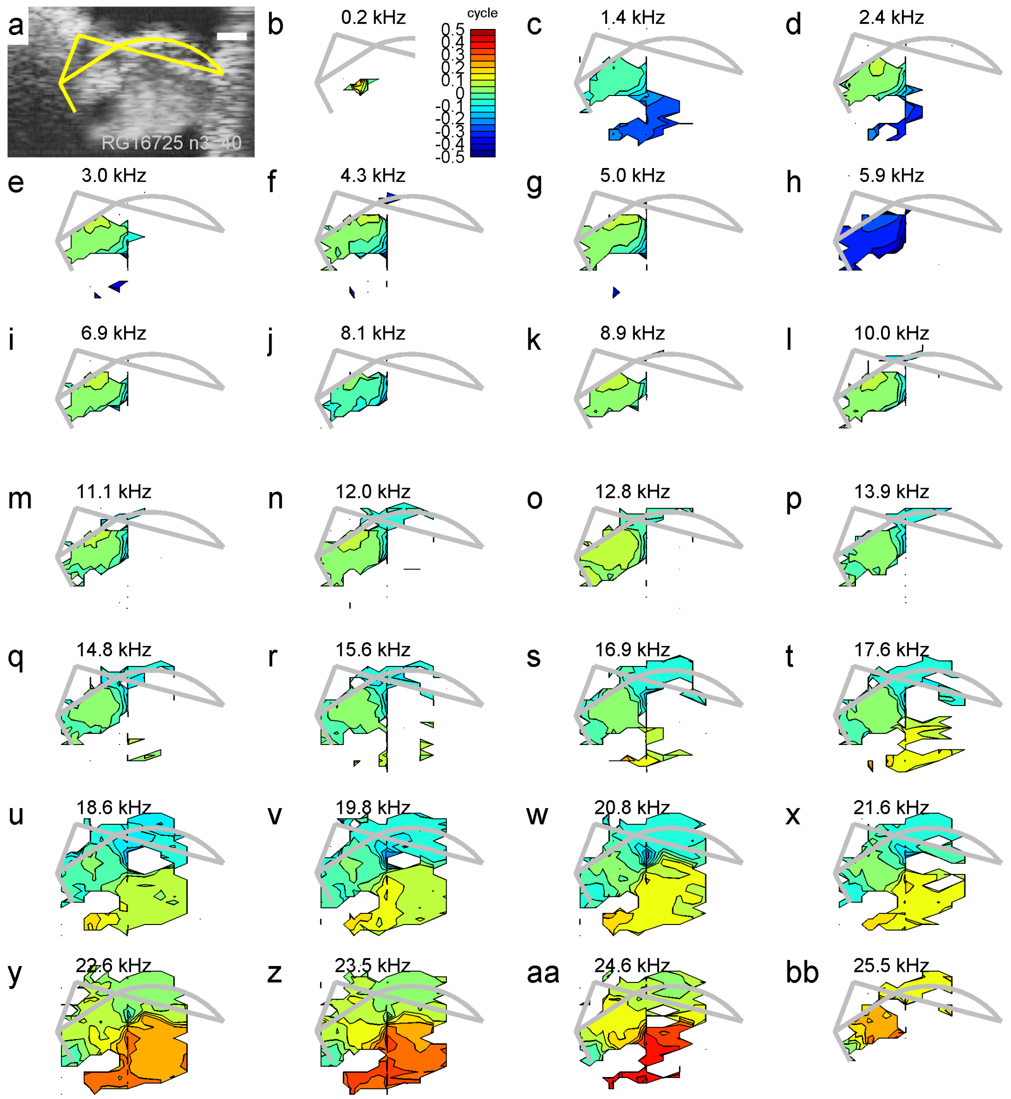

Supplementary Figure 3. Maps of vibration phase for acoustic frequencies spanning 0.2-25.5 kHz (panels **a-bb**) in the same preparation shown in Figs. 2 and Supplementary Fig. 2. Scale bar in panel a, 25 µm. Color code (legend in panel b) indicates vibration phase normalized to the phase observed at the position of the magnitude peak (locations marked by asterisks in Supplementary Fig. 2). CF was 22.6 kHz, sound level 40 dB (see Fig. 2 of main text).

Supplementary Note 2: Frequency dependence of vibration hotspots

Supplementary Figs. 2 and 3 illustrate the frequency dependence of the vibration hotspots that form the main focus of this work. The hotspot becomes more prominent (i.e. more tightly focused, with steeper edges) as frequency increases towards the preparation's CF (e.g. compare the amplitude patterns at 1.4 and 20 kHz in Supplementary Fig. 2). At and above CF, the hotspot becomes less prominent, and gives an impression that its vibration is "leaking out" progressively onto the BM (e.g. from 23.5 to 25.5 kHz). Phase

differences of up to 0.5 cycles are observed between the hotspot and surrounding regions (e.g. the tectal and Hensen cell area ~50 µm lateral to the hotspot) at both low and high frequencies (e.g. 1.4 kHz and >22 kHz; Supplementary Fig. 3).

Supplementary Note 3: Physics of longitudinal motion in surface waves

This note discusses the occurrence of longitudinal motion in surface waves in general and cochlear traveling waves in particular.

Cochlear traveling waves are surface waves, i.e., they propagate on the interface between layers having different material properties. Familiar examples of surface waves are waves on the sea and Rayleigh (seismic) waves. Such waves are never purely transverse or purely longitudinal; they always involve a mix of these two displacement directions[1,2]. This mixed character is particularly inevitable in surface waves on incompressible fluids, because volume conservation prohibits fluid moving in columns in the presence of rigid boundaries.

Importantly, longitudinal and transverse displacements are not in phase, resulting in elliptical orbits of the wave medium. In the absence of friction, one of the axes of the ellipse is aligned with the direction of propagation[1,2], so the longitudinal and transverse components are “in quadrature” (i.e., they have a 0.25-cycle phase difference). Friction typically causes the ellipses to tilt with respect to the propagation axes[3,4], even in isotropic media. Structural anisotropy (which is abundant in the organ of Corti) is another potential source of tilt. Ellipse tilting further complicates the phase relationship between longitudinal and transverse vibration components; it is no longer a simple 0.25-cycle difference. Tilted ellipses are illustrated in Fig. 8a,b of the main text and in more detail in Supplementary Fig. 4a. The exact aspect ratio of the ellipse (ratio of axis lengths) and its tilt depend on wavelength and on details of the propagating medium that are unknown in the case of the cochlea (e.g. anisotropy of both elastic and frictional properties). The incompressibility of the cochlear fluid and the nearby presence of rigid boundaries, however, impose a number of characteristics that may be experimentally tested. These characteristics are derived in Supplementary note 4.

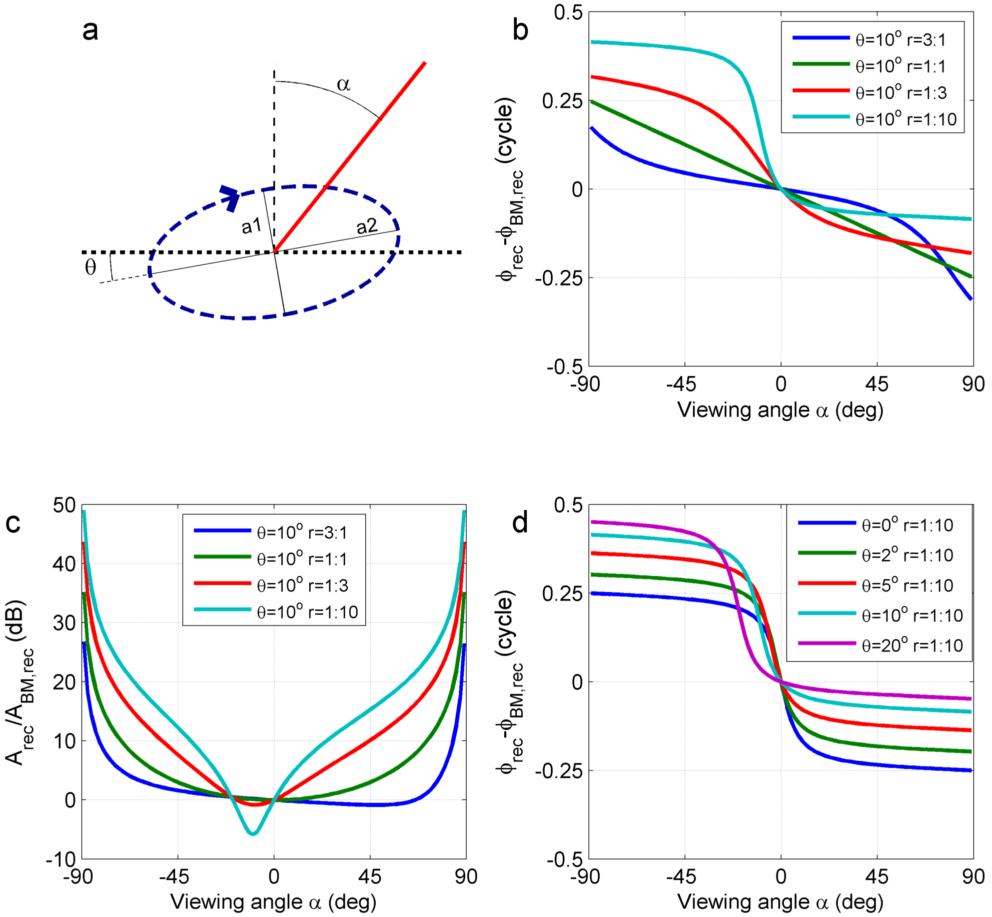

Supplementary Figure 4. Measurable effects of longitudinal wave motion. **a** Diagram of the clockwise elliptical motion (see text), with ellipse axes a1, a2, tilt angle θ and viewing angle α as indicated. The *dotted horizontal line* represents the longitudinal direction as defined by the BM, which is also the direction of wave propagation (from left to right). The *vertical dashed line* is the normal to the BM, which defines the transverse direction. The *red line* represents the measurement beam. **b, c** Dependence of measured phase difference $\Delta\varphi$ and amplitude ratio R_A (see text) on viewing angle α for various values of aspect ratio a1:a2 indicated in the key. Ellipse tilt θ fixed at 10 degree. d Dependence of measured phase difference $\Delta\varphi$ on viewing angle α for various values of ellipse tilt θ. Aspect ratio of the ellipse fixed at 1:10.

Supplementary Note 4: Testing for longitudinal motion in OCT data

This Supplementary Note analyzes the consequences of longitudinal motion for vibration measurements. The analysis leads to the identification of six experimentally testable characteristics of longitudinal wave motion.

From the experimenter's viewpoint elliptic motion causes methodological complications not previously addressed in cochlear mechanics. Elliptic vibration lacks a unique amplitude and phase because the two vibration components along the ellipse axes are in quadrature and generally have unequal amplitudes. The vibration recorded by the

interferometer is the projection of the elliptical motion onto the measurement beam. The recorded amplitude A_{rec} and phase φ_{rec} thus depend on the viewing angle α with respect to the transverse direction and on the tilting angle θ of the ellipse (Supplementary Fig. 4). In formulas:

$$\begin{aligned} A_{\text{rec}} &= A\sqrt{\cos^2(\theta+\alpha)+r^2\sin^2(\theta+\alpha)} \\ \tan\varphi_{\text{rec}} &= -r\tan(\theta+\alpha) \end{aligned}, \tag{1}$$

where A is the amplitude of the vibration component along axis a_1 of the ellipse (Supplementary Fig. 4a); r is the aspect ratio of the ellipse (so rA is the amplitude along a_2, the other ellipse axis); θ is the ellipse tilt and α is the viewing angle as indicated in Fig. 8b and Supplementary Fig. 4a.

Assuming that BM motion is transverse (see Discussion) and matches the transverse component of the nearby elliptical trajectories[3,4], its motion is a projection onto the transverse axis:

$$\begin{aligned} A_{\text{BM}} &= A\sqrt{\cos^2\theta+r^2\sin^2\theta} \\ \tan\varphi_{\text{BM}} &= -r\tan\theta \end{aligned}. \tag{2}$$

Because BM motion is one-dimensional, its subsequent projection onto the measurement beam leaves its phase unaffected and reduces its amplitude by a factor cos α. This yields

$$\begin{aligned} A_{\text{BM, rec}} &= \cos\alpha\, A\sqrt{\cos^2\theta+r^2\sin^2\theta} \\ \tan\varphi_{\text{BM,rec}} &= -r\tan\theta \end{aligned}. \tag{3}$$

for the observed BM motion. We used Supplementary Equations 1 and 3 to compute the predicted dependence on viewing angle α of the amplitude ratio $R_A = A_{rec}/A_{BM,rec}$ and phase difference $\Delta\varphi = \varphi_{rec}-\varphi_{BM,rec}$ (Supplementary Fig. 4b,c). Their dependence on the ellipse tilt is illustrated in Supplementary Fig. 4d. This analysis reveals that the observed phase difference between the hotspot and the BM can exceed 0.25 cycle and approach 0.5 cycle. This means that during a substantial fraction of the stimulus cycle, hotspot and BM move in opposite directions when viewed from an oblique angle.

At first sight this phase opposition seems to contradict the basic assumption, expressed by Supplementary Equation 2, that the BM moves in sync with the transverse component of the hotspot motion. The paradox is resolved by realizing that the observed BM motion involves a double projection: first onto the transverse axis, then onto the measurement beam. The situation is analogous to a sailboat's ability to sail partially against the wind. The wind force is first projected onto the normal of the sail and then onto the longitudinal axis (the keel) of the boat.

As a final step toward an inventory of experimentally testable consequences of longitudinal motion we note that (i) lower frequencies correspond to larger wavelengths; (ii) larger wavelengths produce an increasing dominance of longitudinal motion, causing an increasing longitudinal stretching of the ellipses[1]. Combining this with Supplementary Equations 1-3 and Supplementary Fig. 4b-d, we arrive at the following list of characteristics:

1. Larger phase differences $\Delta\varphi$ occur at lower frequencies;
2. Larger amplitude ratios occur at lower frequencies;
3. Low-frequency $\Delta\varphi$ values may exceed 0.25 cycle and approach 0.5 cycle;
4. When watching the wave "run away" ($\alpha<0$), the hotspot leads BM ($\Delta\varphi>0$)
5. When watching the wave approach ($\alpha>0$), , the hotspot lags BM ($\Delta\varphi>0$)
6. Almost-perpendicular beams (small $|\alpha|$) can still cause $\Delta\varphi$ values that differ substantially from zero.

In the last paragraph of subsection of Results, *Testing for longitudinal motion in the hotspot*, we evaluate whether the data presented in this work comply with these predicted characteristics.

Supplementary References

1. Lighthill, J. *Waves in Fluids*. (Cambridge University Press, 1978).
2. Graff, K. F. *Wave motion in elastic solids.* (Dover Publications, 1991)
3. Lighthill, J. Acoustic streaming in the ear itself. *J. Fluid Mech.* **239,** 551–606 (1992).
4. Marquardt, T. Illustrations of Lighthill's 1992 Equations for the Two-Dimensional Fluid Motion and Associated Viscous Dissipation within the Cochlea. in *To Ear and Back Again: Advances in Auditory Biophysics (edited by C Bergevin and S. Puria)* (AIP Publishing, Melville, NY, 2018).
