# Supplementary material for: Vibration hotspots reveal longitudinal funneling of sound-evoked motion in the mammalian cochlea
Source: Nat Commun. 2018 Aug 3;9:3054. doi: 10.1038/s41467-018-05483-z (PMC6076242; doi:10.1038/s41467-018-05483-z)
Supplement: Supplementary file 3 — Description of Additional Supplementary Files [file 41467_2018_5483_MOESM3_ESM.pdf]

Description of Additional Supplementary Files

File Name: Supplementary Movie 1
Description: Animation of longitudinal motion in the schematized organ of Corti.
